# Combining personal with social information facilitates host defences and explains why cuckoos should be secretive

**DOI:** 10.1038/srep19872

**Published:** 2016-01-22

**Authors:** Rose Thorogood, Nicholas B. Davies

**Affiliations:** 1 Department of Zoology, University of Cambridge, Downing Street, Cambridge, CB2 3EJ, UK

## Abstract

Individuals often vary defences in response to local predation or parasitism risk. But how should they assess threat levels when it pays their enemies to hide? For common cuckoo hosts, assessing parasitism risk is challenging: cuckoo eggs are mimetic and adult cuckoos are secretive and resemble hawks. Here, we show that egg rejection by reed warblers depends on combining personal and social information of local risk. We presented model cuckoos or controls at a pair’s own nest (personal information of an intruder) and/or on a neighbouring territory, to which they were attracted by broadcasts of alarm calls (social information). Rejection of an experimental egg was stimulated only when hosts were alerted by both social and personal information of cuckoos. However, pairs that rejected eggs were not more likely to mob a cuckoo. Therefore, while hosts can assess risk from the sight of a cuckoo, a cuckoo cannot gauge if her egg will be accepted from host mobbing. Our results reveal how hosts respond rapidly to local variation in parasitism, and why it pays cuckoos to be secretive, both to avoid alerting their targets and to limit the spread of social information in the local host neighbourhood.

Individuals often vary costly defences in relation to the degree of danger from predators or parasites^1^, yet how threat levels are assessed remains unclear. This is surprising, given that the perception of risk can have consequences for population structure^2^ and communities^3^,^4^, as well as influencing pathways of coevolution^1^,^5^. Understanding how potential victims perceive risk can illuminate how enemies behave too; for example, if prey or hosts marshal defences in response to enemy cues, it should pay their enemies to suppress these cues.

In theory, individuals might benefit by broadening risk assessment from beyond their own experience of enemies (personal, or private information) to include the responses of others in the local neighbourhood (social, or public information). Individuals are often assumed to trade one source of information against another, with social information being used to update personal information that may be out-of-date, or costly to acquire^6^,^7^. However, animals might rely on both sources to predict the probability of an outcome^8^,^9^,^10^. The key question then, is whether integrating personal and social information about local conditions facilitates the best response.

Hosts of the common cuckoo (*Cuculus canorus*) provide a good model for addressing this question^11^. The female common cuckoo lays just one egg per host nest. Soon after hatching, the cuckoo chick ejects the host’s eggs and young, so it pays hosts to reject cuckoo eggs to save their own brood. However, cuckoo eggs are often mimetic, so clutch appearance alone is an unreliable cue to parasitism and egg rejection entails recognition errors^11^. Nevertheless, long-term studies of reed warblers (*Acrocephalus scirpaceus*), a common host in marshland, show that individuals do reject eggs, and this varies in relation to fine-scale spatial and temporal variation in parasitism risk^12^,^13^,^14^.

This raises the question: how do hosts assess parasitism risk? Reed warblers do this partly by personal observation of local cuckoo activity. When parasitism rates are high, they readily approach and mob adult cuckoos, and the sight of a cuckoo at their nest can increase their propensity to reject eggs^15^,^16^,^17^. However, female cuckoos are secretive and parasitism is rapid (egg laying can involve just a 10 s visit to a host nest^18^,^19^), so looking out for a cuckoo at the nest is costly in terms of time required for surveillance^20^. Furthermore, cuckoos resemble hawks^21^ so close approach is potentially dangerous. These costs of acquiring personal information might explain why a cuckoo at their nest (real or model) does not always stimulate host egg rejection^13^,^17^,^22^.

Recent experiments reveal that several hosts also respond to social information of local cuckoo activity^23^. If reed warblers are alerted to a cuckoo on a neighbouring territory, they are more likely to approach and mob a cuckoo at their own nest^24^,^25^. However, social information might do more than simply lead individuals to copy responses of neighbours^26^; it could escalate other defences too.

Our previous studies suggest that social information may well be important in priming egg rejection. Whereas the sight of a cuckoo at their nest stimulated host egg rejection at high rates of parasitism (20% nests parasitised), it no longer did so when parasitism rates were low (<5% parasitism)^13^. When cuckoos are abundant, hosts will be well aware of the risks of parasitism from frequent personal encounters. Social cues of local cuckoo activity will consequently also be abundant, and information could therefore spread rapidly through local host populations^25^. Conversely, when cuckoos are scarce hosts will rarely encounter them, and so will be much less likely to be primed for egg rejection. Under these conditions, any social cues will be especially valuable in widening the source of information of local parasitism risk.

In this paper we report experiments designed to test whether egg rejection depends on the integration of both personal and social information of local cuckoo activity. Our study was done in two years with low rates of natural parasitism (ca. 5%, see methods). These low rates are similar to those in our previous study where we found no effects of personal information of local cuckoo activity on a focal pair’s egg rejection^13^, and so where we might expect social information to be most effective in amplifying cues of parasitism risk.

## Results

We used previously validated models of adult cuckoos to vary host perception of local parasitism risk, with a harmless intruder (parrot) as a control^12^,^25^,^27^.

### Varying personal and social information by experiment

In each treatment, a focal pair experienced two intruder presentations, at least 2 h apart, and we alternated the order of personal and social information. There were four treatments, assigned sequentially:

(a) Cuckoo on neighbour’s territory; cuckoo at own nest (both social and personal information about local cuckoo activity).

(b) Cuckoo on neighbour’s territory; control at own nest (only social information about local cuckoo activity).

(c) Control on neighbour’s territory; cuckoo at own nest (only personal information about local cuckoo activity).

(d) Control on neighbour’s territory; control at own nest (no information about local cuckoo activity).

After the presentation at the focal pair’s nest, we manipulated one egg in the clutch to simulate parasitism by a cuckoo. We predicted that if either personal or social information alone were sufficient to stimulate defences, focal pairs would reject an egg at similar rates, regardless of whether they observed a cuckoo on their own (c) or a neighbouring territory (b). However, if social and personal information are integrated, then focal pairs who witnessed a cuckoo both at their own nest and on their neighbour’s territory (a) would show the greatest escalation of egg rejection.

**Figure 1:**
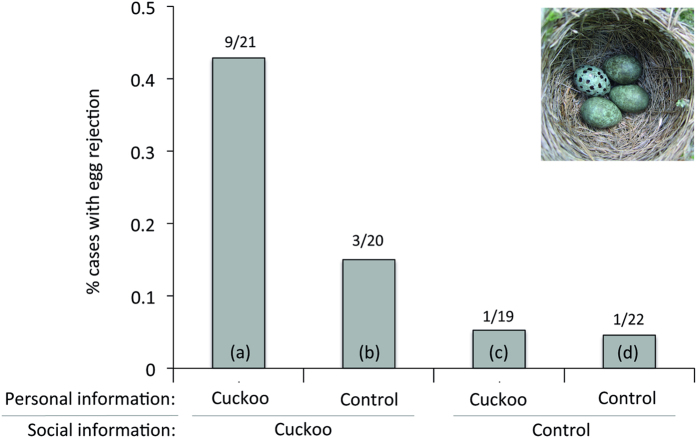
The effects on egg rejection of personal information (intruder on own territory) and social information (intruder on neighbouring territory). There were four treatments: (**a**) social and personal information of cuckoos, (**b**) social information of cuckoos and personal information of control intruder, (**c**) personal information of cuckoos and social information of control, and (**d**) both personal and social information of a control intruder. Number of egg rejections out of the number of focal pairs in each treatment are shown above bars. Only treatment (**a**) was significantly different from (**d**): planned model contrast, *t* = 2.33, *p* = 0.023. Inset: an example of a clutch with an experimental egg (spotted).

Of 82 focal pairs, 11 rejected the experimental egg only, 1 rejected the experimental egg and one own egg, and 2 rejected one own egg (14 rejections in total). The following analyses included both correct rejections (experimental egg) and rejection errors (own egg), to examine the effects of treatment on the focal pair’s threshold for egg rejection (see Fig. 1). The results were the same if we considered only rejection of the experimental egg.

Overall, there was significant variation in egg rejection across the four treatments (GLM likelihood ratio test: χ^2^ = 19.2, *p* < 0.001) and this arose entirely because rejection was highest when hosts had access to both personal and social information about cuckoo activity (treatment (a), Fig. 1). The order of experiencing personal or social information in treatment (a) had no influence on rejection (*t* = 1.35, *p* = 0.20; too few rejections occurred to test for order effects in other treatments).

### Controlling for encountering a cuckoo twice

In a second experiment, we tested for the possibility that the enhancing effect on egg rejection in treatment (a) in experiment 1 arose simply because the focal pair encountered a cuckoo twice, rather than once as in treatments (b) and (c). There were two treatments, again assigned sequentially, with the same protocol as in the previous experiment. This time, each treatment had three intruder presentations, in random order, again with at least 2 h between each one:

(a) cuckoo on neighbour’s territory (plus playback to attract the focal pair); cuckoo and control trials at own nest.

(b) control on neighbouring territory (plus playback); two cuckoo trials at own nest.

We manipulated one egg after the second presentation at the focal nest. We predicted that if social information of local cuckoo activity enhances personal information, egg rejection in (a) should be greater than in (b), and at the same level as treatment (a) in experiment 1. If simply experiencing a cuckoo for a second time elevates egg rejection, then both treatments should have similar high rejection rates.

**Figure 2:**
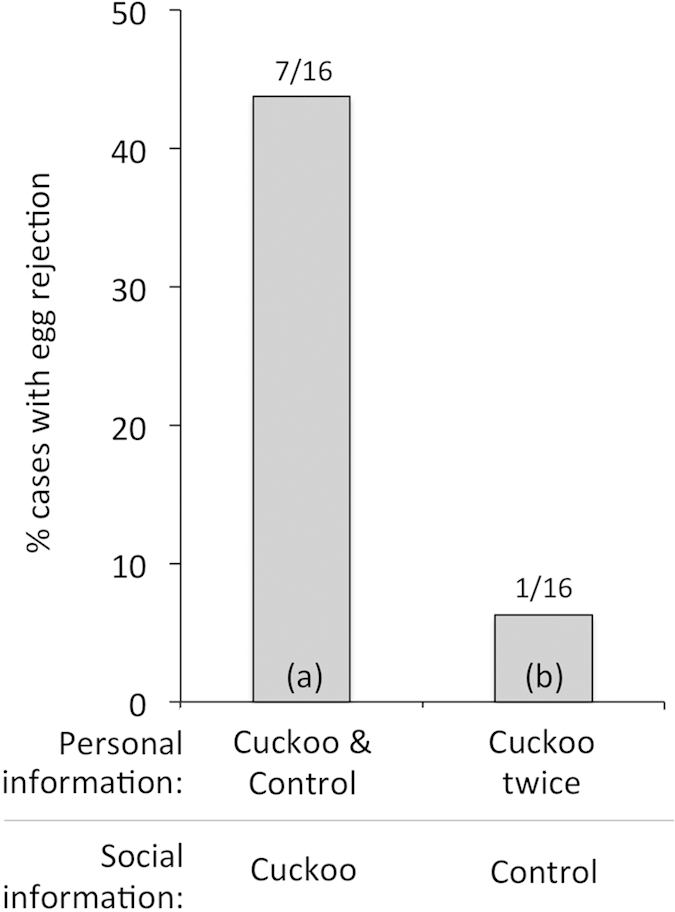
An experiment to control for the effects of seeing a cuckoo twice. Focal pairs in (**a**) witnessed a cuckoo and a control in successive trials at their own nest, and a cuckoo on a neighbour’s territory, and in (**b**) the cuckoo was presented twice at their own nest, with a control intruder at their neighbour’s. Number of egg rejections out of the number of focal pairs in each treatment are shown above bars.

Of 32 focal pairs, 7 rejected the experimental egg, and 1 rejected two own eggs before deserting (8 rejections in total). Egg rejection was still greater in response to both social and personal information of local cuckoo presence rather than only personal information (Fig. 2(a) versus (b); *t* = 2.20, *p* = 0.034). This effect did not differ from that in experiment 1 (comparing treatments (a) from the two experiments; Fisher’s exact test; *p* = 1), and again there was no effect of order of personal vs. social cuckoo presentation on egg rejection (treatment (a), *t* = 1.03, *p* = 0.32).

### Links between host defences

Previous work shows that cuckoos are less likely to parasitize host pairs that mob^12^. Our results here suggest that by doing so, they avoid alerting neighbours. To test whether mobbers are also more likely to reject eggs, we combined data from treatment (a) in both experiments to provide a sample size sufficient for analysis. Our previous work shows that social information of cuckoos promotes mobbing behaviour^25^. Therefore we restricted our dataset to only focal pairs who had the neighbour trial with a cuckoo first (n = 18 pairs, 5 rejections), and so had all received the same cues before measurement of their defences. Pairs that mobbed cuckoos were not more or less likely to reject foreign eggs: 2 out of 10 versus 3 out of 8 pairs who did not mob (Fisher’s exact test: *p* = 0.61).

## Discussion

Our results show that a combination of social and personal information of local cuckoo presence is necessary to stimulate egg rejection. Control intruders had no effect, and personal experience or social cues alone were insufficient to mount this defence. This explains why a cuckoo at a focal nest has strong effects on egg rejection only at high rates of parasitism, when hosts are likely to already be aware of local cuckoo activity, but not at low parasitism rates, where hosts would more rarely encounter cuckoos^13^.

Why was personal information alone not sufficient? Video recordings of natural parasitism reveal that even when reed warblers have witnessed a real cuckoo at their nest, this did not always elicit egg rejection^17^. Personal information alone may be unreliable for two reasons: reed warblers may not be certain that the hawk-like bird at their nest is a cuckoo^21^ and female cuckoos sometimes visit host nests just to inspect the clutch or to remove a host egg, but do not lay^17^. Therefore, the presence of a cuckoo at the nest is not a certain predictor of parasitism.

Likewise, why did social information alone not stimulate egg rejection? Female cuckoos defend laying territories, and they parasitise host nests over a period of about two months, so parasitism of a neighbouring nest increases the chance that a focal pair are in a cuckoo’s territory and will be parasitised too^12^. However, again it is not a sure sign because a female cuckoo does not parasitise every host nest in her territory. Therefore a combination of social information (there is a cuckoo active in the local neighbourhood) and personal information (the cuckoo has found my nest) might be the most reliable cue to parasitism risk. The effect of social information might simply be to reduce the uncertainty that intruders in the local neighbourhood are cuckoos rather than hawks^5^, and so reduce the response threshold for egg rejection^28^. It might also facilitate host learning, enhancing the ability of naïve hosts to distinguish cuckoos from other intruders, and so enable them to act on personal information^5^,^29^.

Our results reveal that pairs that mobbed an adult cuckoo were not more (or less) likely to reject an egg. This suggests that mobbing and egg rejection are independent lines of defence (see also^30^). They might be independent because they involve different cognitive and behavioural skills^30^, and these might be acquired independently. Alternatively, information use may trigger defences independently if the costs of mobbing and egg rejection vary (for example, if the nest environment has different effects on the visibility of eggs or cuckoos). These possibilities deserve further study.

In our reed warbler population, mobbing an adult cuckoo is an effective first line of defence; it reduces parasitism risk by four-fold in high risk sites, namely those with trees and bushes where cuckoos can hide away to monitor host behaviour^12^. Mobbing may sometimes be effective in driving the cuckoo away, because she faces the risk of personal injury from host attacks^31^, but our results suggest another reason. Although mobbers are not more likely than non-mobbers to reject her egg, their calls will alert neighbours to her presence and increase their chances of egg rejection if, in future, she tried to parasitize their nests. Her secretive behaviour and rapid laying are essential, therefore, not only to reduce the chance she alerts the pair at the nest she is targeting, but also to limit the spread of social information of parasitism risk in the local neighbourhood. When hosts nest in high density^12^, this in turn makes mobbing an even more effective deterrent from the host’s point of view.

Our study highlights the importance of distinguishing how information is acquired, and which behaviours are altered in response. Previous experiments have shown that the sight of a cuckoo being mobbed on a neighbouring territory does not always stimulate increased mobbing of a cuckoo by focal birds on their own territory^5^,^24^,^25^. Nevertheless, our results here show that even in non-mobbers this social information can reduce the threshold for egg rejection. Social transmission of information clearly cannot be inferred simply from whether individuals copy the behavioural responses of others. Other studies have shown that perceived risk of predation (via playbacks of predator vocalisations) can release a suite of prey defences^2^,^4^, so translating information across behavioural contexts is likely to be widespread. In nature, individuals have access to information from a wide range of social or personal encounters^8^, so combining and generalising information may be the most effective way to adapt behaviour to local conditions.

## Methods

### Study area

We conducted experiments during May to July 2013 and 2014 on Wicken Fen (52˚18’29′N, 0˚16’50′E) Cambridgeshire, UK. Experiment 1 was conducted in both years, experiment 2 in the second year. Within a year, each pair was tested just once. Given the large population of reed warblers on our study site (only 26% of c.300pairs tested in 2013, and 10% in 2014), the wide area over which we did experiments and annual adult survival of 33 to 52%^32^ it is unlikely that the same pair was tested again in the second year. Natural parasitism rates did not differ between years (2013: 10 out of 195 nests parasitized, 5.1%; 2014: 11 out of 194 nests, 5.7%), and neither did egg rejection (experimental eggs rejected at 13 out of 74 nests in 2013 and at 9 out of 40 nests in 2014; Fisher’s Exact test: *p* = 0.62). These low rates of natural parasitism are similar to those in our previous study where we found no effect of personal information alone (a model cuckoo at their nest) on a focal pair’s egg rejection^13^.

### Varying personal and social information by experiment

We used models of two intruder stimuli: an adult cuckoo (grey upperparts, pale underparts with barring) and a harmless intruder as a control (grass parrot; dark green upperparts, pale green underparts), which were validated in previous studies^12^,^25^,^27^. Models were made from painted balsa wood and were identical in length (33 cm) and in the shape of body and tail. We have shown in previous experiments that reed warblers rarely mobbed a parrot model, but they often mobbed a cuckoo model and their response was strongly correlated with that to taxidermic cuckoo mounts, and similar to their response to a live cuckoo^25^,^27^. We used two, virtually identical, models of cuckoos and parrots, which did not differ in their effects on reed warblers’ mobbing and egg rejection responses^12^,^25^,^27^. We performed experiments only on naturally unparasitised nests, on the day the focal female laid her fourth egg (usually the final egg of the clutch).

Personal information: we placed the model (either cuckoo or parrot) adjacent to, and touching, the rim of the focal pair’s own nest. From c.10 m away, we recorded the total number of rasp vocalisations and bill snaps (both male and female mob and this measures their response^27^). Trials lasted for 10 min after the first approach.

Social information: the model (either cuckoo or parrot) was placed on the neighbouring territory (<40 m from focal nest) and accompanied by broadcasts at constant amplitude (95 dB at source) of reed warbler mobbing signals (rasp calls and bill snaps). Playbacks were one of five digital recordings of a natural mobbing response to a taxidermic cuckoo mount, recorded previously from different birds^27^ and we used them in sequence for successive experiments. Rasp calls and bill snaps are not given specifically in response to cuckoos, but they are given at a much higher rate than to other intruders^27^. The playback was broadcast for 10 min, ample time to attract the focal pair from an adjacent territory up to 40 m away^27^. Observations of colour-ringed birds show that usually only next-door neighbours are attracted by mobbing, not birds from more distant territories^20^,^27^. In cases where neighbours had a nest, these were at various stages of incubation or with chicks up to 7 days old. The model was then placed adjacent to and touching the rim of the nest. If neighbours had not begun to nest, the presentations were done in the centre of the territory. The stage of the neighbour’s breeding cycle occurred randomly among treatments and had no effect on focal pairs’ egg rejection (outcome of both experiments combined: *p* = 0.48).

While the playbacks simulated social information that there was an intruder at large, and most likely a cuckoo (from the high vocalisation rates), we cannot know for sure whether other aspects of the neighbour’s behaviour (displays, vocalisations, agitation^27^) might affect the focal pair’s response. As in our previous experiments^5^,^25^, neighbours approached their nests as soon as the playbacks began and hopped about close by throughout the trial. It was difficult to record their vocalisations during the playback itself, so we recorded their number of bill snaps and rasp calls for one minute after the playback had ended. There was no difference in the neighbours’ vocalisations between the two treatments that provided social information about cuckoos in Experiment 1 (treatments (a) and (b), Fisher’s Exact test, *p* = 0.33), and there was no effect on focal egg rejection behaviour (change in model fit when term included in analysis: χ^2^ = 1.30, *p* = 0.21). Therefore, it is likely that any neighbour’s vocalisations were swamped by the effects of the playback. Nevertheless, we are confident that our presentations simulated social information, because without mobbing calls focal birds would not have visited the neighbour’s territory^20^.

We alternated whether focal pairs received personal or social information treatments first, and immediately after the model presentation at the focal pair’s own nest, we simulated cuckoo parasitism by painting one of their four eggs with forty small brown spots using a Staedtler permanent felt tip pen (Lumocolor® 314-7). Three days later, we recorded any egg ejections or clutch desertions.

All experiments were conducted in accordance with protocols approved by Natural England.

### Statistical analyses

We used generalised linear models where egg rejection was modelled according to a quasibinomial error distribution (data were underdispersed) with a logit link. Significance of overall terms was assessed using likelihood ratio tests based on a χ^2^ distribution, and comparisons among treatments used *t*-statistics from model contrasts. In each analysis we included the covariate of date, as previous experiments have demonstrated that rejection declines over the season in our population^13^. To compare results of experiments, we used Fisher’s Exact tests. All statistics were performed in R version 3.0.2.

## Additional Information

**How to cite this article**: Thorogood, R. and Davies, N. B. Combining personal with social information facilitates host defences and explains why cuckoos should be secretive. *Sci. Rep.* **6**, 19872; doi: 10.1038/srep19872 (2016).
